# Informing patients with progressive neurological disease of their health status, and their adaptation to the disease

**DOI:** 10.1186/s12883-019-1488-y

**Published:** 2019-10-25

**Authors:** Radka Bužgová, Radka Kozáková

**Affiliations:** Department of Nursing and Midwifery Faculty of Medicine, University of Ostrava, Syllabova 19, 700 30 Ostrava, Czech Republic

**Keywords:** Adaptation, Communication, Needs, Progressive neurology diseases, Quality of life

## Abstract

**Background:**

Progressive neurological diseases, such as multiple sclerosis, Parkinson’s disease, Huntington’s disease, significantly interfere with patients’ lives, and those of their families. The aim of the research was to establish whether the extent of the information on patients’ health conditions, and the way patients learn this information from doctors affect their adaptation to chronic and progressive diseases.

**Methods:**

Qualitative methodology was used for a total of 52 participants (patients with progressive neurological diseases, their family members, and health and social workers). Data were collected using individual, in-depth interviews and focus groups. Analysis of data for interpretation, conceptualization, and re-integration was performed by open, axial, and selective coding.

**Results:**

It was determined that adequate information about patients’ health status, and the use of coping strategies are related to their adaptation to their disease, and consequently, to their quality of life. The participants often considered the extent of the information provided, and the way they were informed to be inadequate. Receiving the diagnosis, the progression of the disease, and the end of life were found to be the most burdensome.

**Conclusion:**

Our results show that Czech neurologist should develop better communication skills, particularly for informing patients with progressive neurological diseases. Open communication, emotional support, and support in selecting effective coping strategies can help patients adapt more readily to their disease, and improve their quality of life.

## Background

Progressive neurological diseases (PND), such as multiple sclerosis (MS), Parkinson’s disease (PD), and Huntington’s disease (HD), significantly interfere with patients’ lives, and those of their families [1]. These neurodegenerative or demyelinating diseases cause invalidity, and chronic progressive symptoms; sometimes also affecting the young and those of working age. The combination of movement, cognitive, emotional, and behavioral disorders is usually the result of a pathological action with variable progression [2]. Informing patients about the disease at the very moment the diagnosis has been made can help them gain some control of their situation. Adequate information and emotional support provided by healthcare professionals can have a decisive impact on the disease management process [3].

The Czech law requires from a healthcare provider to ensure that patients are adequately informed about their health status, and about the individual care plan proposed and the changes it will involve, in a comprehensible way. In extraordinary cases, doctors are permitted to “withhold” this information if it is considered to be for the benefit of the patient, and if this information is not explicitly requested. The way patients are informed about their diagnosis and related health conditions can play a key role in creating a doctor-patient relationship [4]. Informing patients about their diagnosis as soon as it has been made is appropriate, since patients who have a realistic picture of their disease and its progression can plan their future from the first moment the existence of the disease in more than a suspicion, and discuss the support they will need at advanced and terminal stages of the disease [2]. Little research has been conducted on how patients with individual diagnoses are informed of their diagnoses. Boersma et al. [5] examined patients with PD, revealing that patients did not have sufficient opportunity to ask questions. They wanted to know what their diagnosis meant, and who they were supposed to turn to for help and support. Patients felt as if they had been abandoned by their doctor. Lode et al. [6] found that patients with MS who were adequately informed about their diagnosis as soon as it had been made better adapted themselves to the disease. Solari et al. [4] surveyed the experiences that patients with MS had had regarding communication of their diagnosis, revealing that many patients complained about lack of support and communication. The way they were informed about the disease was associated with how well they coped with it.

Research has identified other unmet psychosocial needs in patients with PND [7]. One of the main topics from a recent study into PND [8] that emerged from the data was the lack of awareness of the disease, and how this affected the process of adaptation to the disease. As a result, we decided to explore this concept further. A new research question was defined: “*Does the extent of the information on patients´ health conditions and the way patients with chronic progressive neurological disease learn this information from doctors affect their adaptation to their disease?”*

Researchers have not devoted much attention to the topic to date. Some authors [9, 10] have investigated patients´ adaptation to chronic disease, but in relation to their lack of self-sufficiency. Kim et al. [11] created a theoretical model of psychosocial adaptation to chronic disease, based on a literature review, describing five constructs: courage, practical wisdom, commitment to action, integrity, and emotional transcendence.

## Methods

Qualitative methodology was used to conceptualize patterns of information and adaptation to the disease of patients with PND. This qualitative analysis takes into account the event being studied (adaptation to the PND disease), the individual meanings made of it (views of patients, family members, and health care professionals), and the broader social structures (hospitals, and health and social facilities) [12].

### Sample

A total of 52 participants took part in the qualitative research. The sample was made up of 11 patients with PND, six family members of patients with PND, and 35 healthcare professionals (six medical doctors) working with patients with PND (see Table. 1). The selection of participants was intentional, and made according to the following criteria:

1. Participation limited to patients with selected PND (MS, PD, atypical Parkinsonism, HD), at least one year after diagnosis, at less 10 years after diagnosis, age > 18 years, Mini Mental State Examination MMSE ≥24 points; able to participate as determined by the research coordinator (RK).
2. Family members of patients with selected PND at least one year after diagnosis, age > 18 years who provided major support for patients, and had direct contact with patients. The family members´ average length of care was 5,4 years (min: 2 years, max. 9 years).
3. Healthcare professionals – with professional competence and experience of care of patients with PND of at least one year in hospitals, health or social facilities, or home care. Ideally, participants should reflect a range of ages, levels, professions (e.g., medical doctor, nurse, social worker, physiotherapist, ergotherapist, psychologist, hospital chaplain) and patterns of working.

**Table 1:** Characteristics of participants

Methods	Participant	Disease or type of profession	number	Age	Gender
Interview	Patients	Multiple sclerosis (MS)	4	36–67	2F, 2 M
N = 20	*N* = 11	Parkinson’s disease (PD)	4	69–82	F, 3 M
		Atypical Parkinsonism (AP)	3	68–73	2F, M
	Family members	Multiple sclerosis (MS)	2	36–82	2F
	N = 6	Parkinson’s disease (PD)	3	51–69	2F,M
		Huntington’s disease (HD)	1	69	F
	Professionals	Medical doctor – neurologist	1	42	F
	N = 3	Social worker for neurology clinic	1	48	F
		Nurse – care for patients with HD	1	41	F
Focus groups	Professionals	Medical doctor – neurologist	3	41–64	F, 2 M
N = 4	*N* = 32	Medical doctor – palliative medicine	1	57	F
		Medical doctor – rehabilitation	1	54	F
		Nurse – long term care	4	29–58	4 F
		Nurse – hospital	4	29–64	4 F
		Nurse – community care, ambulance	7	27–42	7 F
		Physiotherapist	3	26–45	3 F
		Ergotherapist	2	29–39	2 F
		Social worker	3	41–42	3 F
		Psychologist	2	35–40	2 F
		Hospital chaplain	2	45–68	2 M

Legends: F-female, M-male

Professionals, family members, and patients were contacted in all regions of the Czech Republic by the research coordinator. The method of obtaining the sample was the snow ball technique. First respondents were recommended by the head of the university hospital neurology clinic in Ostrava. The remainder of the participants were selected according to recommendations from the studied individuals. Emphasis was placed on participants’ experience of the specified topic. Participants were accepted until theoretical saturation of the sample was achieved (theoretical sampling = data collection based on emerging hypotheses from the ongoing analysis). Potential participants were contacted directly by RB (health professionals) and RK (patients, family members) by email or phone and those who agreed with cooperation were invited to participate in a semi-structured interview or focus group discussion. Recommended participants were selected and addressed randomly in order to achieve theoretical saturation of sample. To meet the needs of theoretical sampling, and achieve theoretical saturation, diversity was sought in experience of PND disease (patients, family members), and in experience of care of patients with PND (healthcare professionals): i.e., disease type, age, location, and number of years making diagnoses or caring for people with PND. Diversity was also sought in type of facilities (hospitals, nursing homes, social facilities, rehabilitation facilities, home care) and their size and location. The intent in seeking such diversity was not to obtain a representative sample, but rather to fit the emerging theory to the data through theoretical sampling.

### Data collection

The data were collected using individual, in-depth, semi-structured interviews (*n* = 20), and focus groups (*n* = 4; 32 professionals). Data collection ceased when theoretical saturation was achieved. We defined saturation as the point in coding when we found that no new codes occurred in the data. There were mounting instances of the same codes, but no new ones.

Individual interviews were conducted with patients with PND and their family members, and with those health professionals who could not participate in the focus groups (*n* = 3). Interviews were conducted in a suitable room, without the presence of others. After explaining the aim of the research and obtaining the participants’ informed consent and a completed demographic form, interviewers asked their questions. The interview guide questions focused on the following experiences: a) problems in the first stage of disease (making diagnosis, provision of information regarding health status, disease and prognosis, provision of information about support and palliative care); b) impact of the disease on patients´ (and family members´) lives; c) problems during this period (physical, social, psychological, and spiritual issues); and d) preferences and opinions regarding end of life care. The participants spoke freely on the topic, and the interviewer then asked them questions based on their responses. Interviews were conversational in style, using open-ended questions from an interview guide (versions for professionals, patients, or family members). The length of the individual interviews was 30–70 min, with a median time of 56 min.

Other healthcare professionals discussed their experience of the topic in a focus group, which lasted 120 min. Written informed consent was obtained at the start of each group, following further explanation of the study by the researchers, and an opportunity for participants to ask questions. The focus groups were facilitated by a researcher (RB). The focus group guide questions focused on the following views and experiences: a) the needs and most frequent problems of patients with PND and their family members, from diagnosis to end of life care (in the first stages of disease – receiving a diagnosis, informed provided about health status, disease and prognosis, information provided about support and palliative care, impact of the disease on patients’ (and family members´) lives; in advanced stages of disease; at the end of life), b) the possibility of applying palliative care to patients with PND, and c) the application of the model of neuropalliative care in the Czech Republic. The focus group guide questions were thoroughly discussed and refined by the research group in advance. The focus group/interview guide was adapted accordingly for the subsequent follow-up discussions/interviews based on the previous responses and ongoing data analysis. All four group sessions took place at the University of Ostrava, Faculty of Medicine, with each focus group consisting of seven to ten participants.

The reason for using both methods (interviews and focus groups) was to obtain as much in-depth information as possible from the smaller group of people involved in the focus groups. This enabled us to understand the context behind the answers given in the group survey and helped us explore topics in more detail than would otherwise be possible in an individual survey alone.

### Data analysis

The discussions and interviews were recorded by a voice recorder, transcribed verbatim and anonymized to ensure confidentiality. On the basis of the initial analysis, three main themes were selected, and then analyzed individually: 1. unmet needs of patients with PND [13], 2. unmet needs of family members [14], and 3. provision of information to patients and adaptation to the disease. This article describes the analysis of domain 3 only.

Analysis of data for interpretation, conceptualization and re-integration was performed by open, axial and selective, according to Strauss and Corbin [15]. Theoretical saturation was considered to be met when the identified themes were robust, and no new codes emerged from the data.

For open coding, so-called meaning units were created [16]. Two researchers (RB, RK) independently codified the raw transcript. First, we conferred regularly to discuss coding, analysis and data interpretation. This was an iterative process, moving between the data and the analytical concepts to develop codes and concepts grounded in the data.

During open coding, we created various terms. We selected a semantic unit as the coding unit and then we coded the transcribed interviews line by line. After we had discussed their interpretations, a coding frame was created. Subsequently, we categorized the codes by means of the constant comparative method, and we named it. Next, we recorded the characteristics of the codes, and the dimensions of the characteristics.

**Fig. 1:**
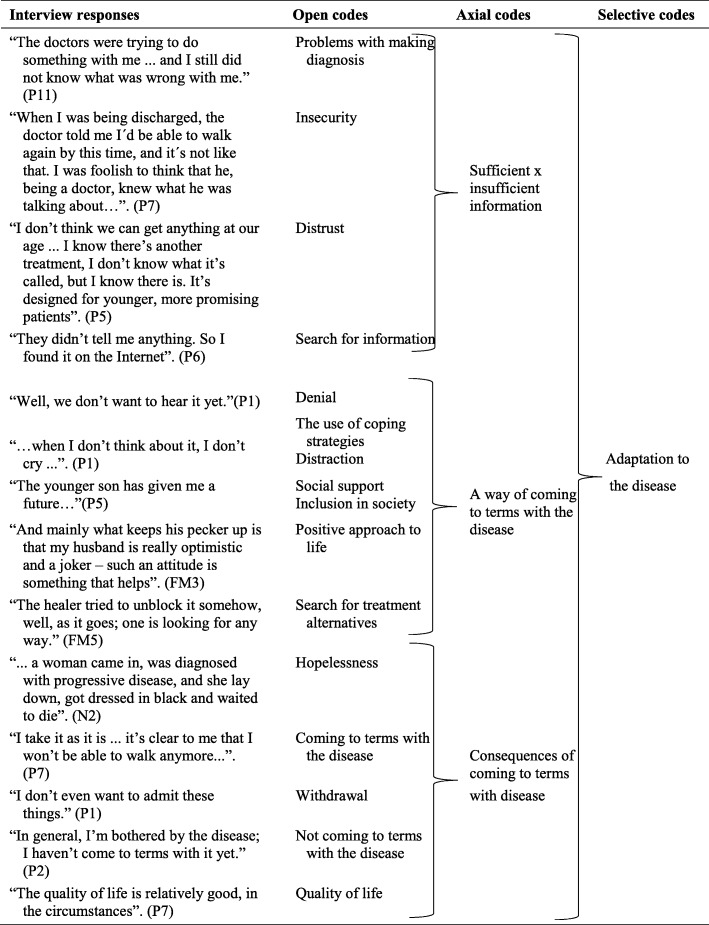
Coding process

For axial coding, we created links between categories and subcategories, within and across individual chapters. Then, we organized the categories into a paradigmatic model according to Strauss and Corbin [15]. The paradigmatic model was completed in a circular, rather than a linear fashion. The possibility of circularity is explicitly permitted by Straus and Corbin. When engaged in axial coding, Strauss and Corbin [15] apply a set of scientific terms to make links between categories visible, and we used a similar organizational scheme: 1) conditions, circumstances, or situations that make up the structure of the studied phenomena (making a diagnosis); 2) actions/interactions, and participants’ routine or strategic responses to issues, events, or problems (sufficient or insufficient information, denial, the use of coping strategies, the search for treatment alternatives, hopelessness); and 3) consequences, and outcomes of actions/interactions (quality of life). Figure 1 depicts a portion of the diagrammatic trail showing how first-level (open) and second-level (axial) codes were compared and consolidated to generate the theme “Adaptation to the disease”.

After the main categories had been obtained, we performed the third selective coding, and we determined domains (the highest level of abstraction). Selective coding included the coding and recoding of particular data based on central concepts from the ongoing analysis. Finally, we created the central category of the investigated material, which became the center of the hierarchical network of categories (adaptation to the disease).

Each statement used in the text is marked by the participant’s number and their role/profession (P - patient, FM - family members, MD – medical doctor, N – nurse).

## Results

**Fig. 2:**
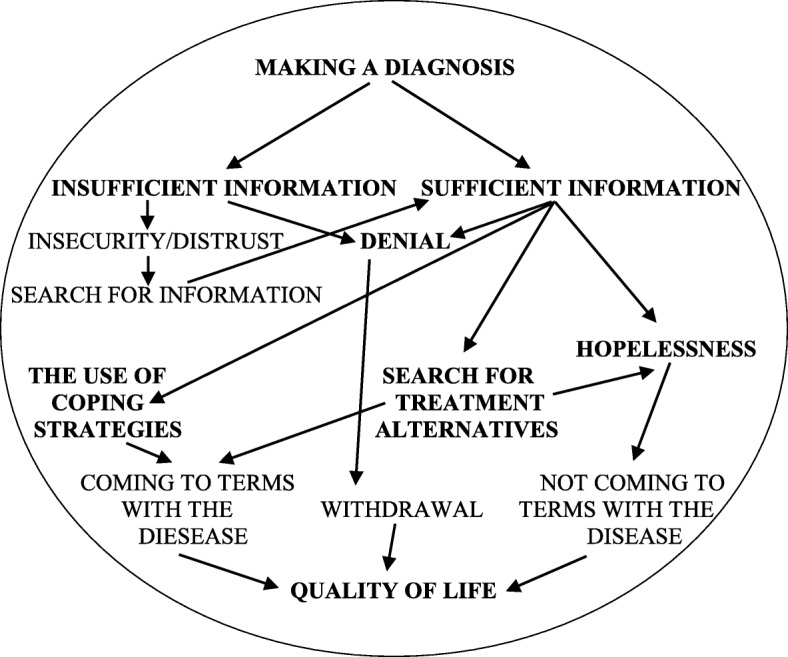
Final Model of Data Analysis

Data analysis resulted in the development of an association between provision of information to patients with PND regarding their diagnosis and health status, and their adaptation to the disease. The category “*making a diagnosis”* was determined as a causal condition for qualitative analysis, leading to the aspect of “*adaptation to disease”*, whose interventional condition was “*sufficient*/*insufficient information”*. Coping strategies and interactions were “a *way of coming to terms with the disease*” (the use of coping strategies, denial, searching for alternative treatments, etc.), resulting in either “*coming to terms with the disease”* or “*not coming to terms with the disease.”* Patients’ quality of life (Fig. 2) was selected as the central category. The basic analytical narrative was formulated as follows: adaptation to disease is a process that is significantly related to how patients with PND experience quality of life. It is conditioned by having sufficient information about the disease, and the use of coping strategies.

At the time of diagnosis, and throughout the course of the disease, some patients received adequate information, while others did not. Although is sometimes difficult to diagnose a PD or MS above a level of suspicion and in case of clinical diagnoses it takes (necessarily) time, lack of information is for the patient a problem. Lack of information led to patient denial of the disease, or to feelings of uncertainty and distrust of doctors. Patients often resorted to searching for information on the Internet or in self-help groups. Those who received adequate information, either from a doctor or from their own research, were able to apply coping strategies and come to terms with the disease, or looked for treatment alternatives. Some patients entered the denial phase or experienced hopelessness, and were unable to come to terms with their disease. Patients who denied the reality of their disease did not wish to plan their future or even consider it. The way the patients coped with the disease affected their quality of life.

The results in each category are described below.

### Making the diagnosis

Being diagnosed with a serious chronic disease is always a turning point in the life of a patient. Some patients learnt of their disease immediately. However, others had to undergo various repetitive examinations before receiving a correct diagnosis from their doctor.

For some patients, there was even a change in the diagnosis: “*Last year I was diagnosed with something completely different. They just did not know what was wrong with me.”* (P3).

Family members reported that doctors from non-specialized health facilities showed a lack of knowledge: “*We had the problem that the doctor did not make the correct diagnosis. He did not point out the problem and cooperate with us ... he was dealing with something else, not with the main thing.”* (FM6).

### Lack of information about the disease

Patients and family members often did not receive adequate information either at the time of diagnosis or at later stages of the disease. Few participants were satisfied with the amount of information provided.

Several participants reported that doctors did not give them any information about the disease: *“They did not tell me anything at all.”* (P5). Patients informed of their disease only by medical report were dissatisfied: “*A doctor who writes a diagnosis and does not tell me what it actually means … He would not even have told me … if I hadn’t read it in the report ... I think it’s inadequate … they should have explained it to me and told me what to expect.”* (P1).

Doctors and other healthcare professionals spoke about the difficulty of giving patients their diagnosis, and informing them about the disease and prognosis at different stages. While doctors find it difficult to give patients such a diagnosis and further unwelcome news that accompanies it, at the same time, they are aware of the consequences of not doing so: “*It’s natural - no one wants to be a prophet and give the worst news. And, moreover, to do so on a daily basis ... but on the other hand, if the patients do not know it, they will never be able to accept it.”* (MD2). Other healthcare professionals had similar opinions: “*If we can’t stand bad news, or we can’t give it, how can we be supportive?*” (N3).

It is particularly problematic when necessary to inform patients or their family members that their health status has deteriorated, or that s/he has progressed to an advanced stage of the disease that is incurable: *“First, we do not even know when to say it. Yeah, we feel it a little bit, but every doctor is different. You don’t learn this at school .... Today, there’s a belief that medicine can cure all diseases, and that doctors prolong life.”* (MD4). Healthcare professionals do not often communicate openly with patients, and are not aware of the kind of information patients would like to receive: “*This is exactly what we healthcare professionals often can’t do. We can’t even ask the patient about what they really want to hear.”* (MD3).

Some patients could not talk openly about their disease with their family, or about the follow-up support they required, because they had not been given sufficient information about the disease: “*We haven’t talked about it yet. Because I only know what I read on the Internet.”* (P2).

Lack of information often made patients **feel insecure** and **begin searching for information** for themselves. They felt a distrust of doctors who did not provide adequate information: “*The doctor doesn’t know … they don’t have enough information about it ... I’ve seen it a hundred times. ... They should be at least a little aware of what it [the disease] means.”* (P11). Some patients lost all respect for doctors: *“When I was being discharged, the doctor told me I’d be able to walk again by this time, and it’s not like that. I was foolish to think that he, being a doctor, knew what he was talking about. He didn’t.”* (P7)*.*

Furthermore, general practitioners may not have sufficient experience of neurological diseases: *“The GP practically does not know what the disease is like. He said he had no experience of it, and he doesn’t know how the disease will progress.”* (FM4).

Patients feel insecure not only when they do not have adequate information about the disease, but also when they meet other patients with the same disease: *“We’re waiting in the waiting room all together. I meet patients, who are much younger than me, and I can see that it [the disease] has such a rapid progression.”* (P4).

Some patients even reported that their doctor advised them not to **look for information** about the disease: *“Doctors tell you not to read it, right? But when they don’t tell you anything, what should we do?”* (P10).

Some patients later received information, for example, from another doctor rather than the one who had first diagnosed and treated them: *“It was the doctor here [in rehabilitation] who suggested that it would get worse. My husband wasn’t told anything either* [crying]. *I think it’s wrong. Because I think we should know it.”* (P9).

Some participants received information from friends: “*Because we had, thanks to my niece, who is a neurologist in Prague, access to information that we’d otherwise never get.”* (FM6). *“When I don’t know anything, I’ll ask my friend with MS or I’ll search for it on the Internet. No one in the hospital will tell me.”* (P3).

### Sufficient information

Some participants were satisfied with the extent of the information provided: *“When I ask the doctor about anything, he’ll answer every question for me. I don’t have a problem.”* (P7). At least one patient positively evaluated communication with a doctor via the Internet. Patients also received the information they required through self-help groups. Some participants mentioned events organized by non-profit organizations, also attended by doctors, who could provide information in an informal setting.

Having sufficient knowledge about the disease enabled some participants to understand its severity: “*We know for 100% that she has realized it from the beginning ... she knew she was going to die.”* (FM4). Another patient reported: *“Well, the doctor said it would be tough, that we should get ready, that it would be very hard.”* (P8).

### Denial

Some patients denied the reality of their disease, despite the information they had received. Although well informed about their illness, either by a doctor or through their own research, they were not willing to contemplate the future: *“Well, we don’t want to hear it yet.”* (P1).

In some cases, they did not wish to know about the severity of their health condition: *“If I went to see a doctor and he would be telling me some options, I’d just want to hear the first one - that I’d get better …*” (P3).

They sometimes hoped for a breakthrough in treatment in the future: *“Two years ago, I was told that it would only get worse and worse. Yes, of course, doctors shouldn’t lie to a patient ... but also the neurologist knows that the brain is so unexplored that anything can change.”* (P2).

Some said they did not want to attend self-help groups to avoid seeing how other patients´ health conditions had deteriorated: *“I can’t go there ... When I see them, some are already in the wheelchair, almost everybody, and I know I’ll be in a wheelchair too ... Because I know that the guy I knew, he’d been walking and when I came back in a year, he was already in a wheelchair. He just couldn’t [walk] at all.”* (P4)*,* or how their relationships with others might deteriorate: *“It’s sad because sometimes the course is very dramatic. Sometimes it ends with the breakup of families. Unfortunately, it isn’t always possible to cope with the psychological stress.”* (P3). The participants found meeting people with the same illness as themselves traumatic: “*I can see some patients that go there, and I am scared. I’m afraid. I’m afraid I’ll end up like the others. I don’t want to. I’ll live my way as long as I can.”* (P6).

### The use of coping strategies

**Distraction** was mentioned as an effective coping strategy by participants: “*What can I do? I have to take it as it is. Crying won’t help me ... When I don’t think about it, I don’t cry.”* (P1). Work helped distract one patient from her condition: *“I tutor kids at home, which helps me. Sometimes I feel like I’m tired or something ... I say to myself, I can’t cry, I can’t lie in bed.’ You have to do it for the kids’ sake - it’s not their fault.”* (P7).

Patients also regarded **social support** and **inclusion in society** as effective coping strategies: *“The younger son has given me a future. He said: ‘I paid for a newspaper subscription for you,’ so that I could be in touch with the world through the daily press. ‘And I paid for a season ticket for the theatre for you.’ … I like the theatre … and I’m glad because it’s a chance to dress up nicely, to get myself in the right mood, and I was going to say that I’ll walk out the door, but I’m not going to walk, I’ll be in a wheelchair* [crying slightly].*”* (P5). Friends also provide support for patients: *“When I fell ill, there were two things that stopped it from getting worse so quickly. When I told my employer about the disease, he told me if I wanted to keep on working, he’d let me ... The second was that at the time I was also working as a youth sports instructor. When I talked to my colleagues, they told me they needed a referee, someone who’d help out … so I could keep on working.”* (P2).

A **positive approach to life** was found to be another coping strategy. This was particularly highlighted by patients’ family members: *“Her only stroke of luck, a huge stroke of luck, was that she was extremely positive. It (HD) has terrible, horrible stages, when you don’t know what it’ll do to you - despite all that she was positive.”* (FM4). A similar experience is described by the wife of a patient with MS: *“And mainly what keeps his pecker up is that my husband is really optimistic and a joker – such an attitude is something that helps.”* (FM3). Other family members described their loved ones as fighters: “*He’s a fighter, he does really want to fight,”* (FM2), or as being patient: *“He possesses a great personal characteristic, that he’s extremely patient. He’ll try to say something ten times, he’ll read a book and maybe it will take him five minutes to turn the page. I’d give up … but he’s patient.”* (FM1).

One of the patients mentioned their faith in God: *“... a belief in God is very important to me, and the sense that health isn’t the most important thing in life helps me.”* (P2).

### Search for treatment alternatives

A number of participants had tried alternative treatments, which, however, were described as unsuccessful: *“The healer tried to unblock it somehow … You look for any way. However, we didn’t find anything that improved his health condition … and in two years his condition has got so much worse.”* (FM5). Other family members also mentioned the search for alternative treatment options: *“Of course, if you’re living with the person for nearly 50 years, you clutch at straws. Someone who was always healthy, who was never ill, never, and who was a great sportsman, so I was clutching at everything.”* (FM2).

### Hopelessness

The severity of progressive disease led some patients to despair: *“The muscles will gradually weaken, that is, I won’t be able to either talk or swallow, I won’t be able to do anything ... then I won’t be able to move at all. It’s no sort of future,”* (P11), or to experience a sense of inferiority: *“You feel like you’re inferior [crying]. You fall ill and you don’t even know why. Then you are no good to anyone anymore,”* (P2); or psychological resignation: *“A woman came in, was diagnosed with progressive disease, and she lay down, got dressed in black and waited to die.”* (N2).

### Coming to terms with the disease

Some patients reported that they had come to terms with the disease: *“I take it as it is ... It’s clear to me that I won’t be able to walk anymore.”* (P7). Another patient described it as a process: *“When I compare it with the beginning, with those years when I could not understand it, and I kept on asking what caused the disease ... So I feel a little more satisfied now. Or I’ve come to terms with the disease.”* (P5). A caregiver for a patient with HD revealed that the patient knew about the severity of their disease: *“‘I’m going to die; I was given 10 years to live.’ So she knew it all the time until the end that she was going to die, that there is an end.”* (FM4).

### Not coming to terms with the disease

However, a number of participants had not come to terms with the disease: *“In general, I’m bothered by the disease; I haven’t come to terms with it yet.”* (P2). They do not often name their disease, but refer to it impersonally as “it”. One patient with MS expressed her wish to be healthy: *“I’d like to be like a normal person, to get up in the morning, go to work.”* (P1).

Some patients who had not come to terms with their disease had unrealistic expectations regarding it: *“I expect that my health condition will improve.”, “I hope it [deterioration] won’t happen to me.”* (P3). Some patients bargained with the disease: *“I hope that I’ll go, if not skiing, so at least cross-country skiing* [crying]. *But I won´ t give up, I won’t.”* (P6).

Family members had had similar experiences: “*He’s trying; he’s incredibly diligent, he still believes that he’ll simply walk off the disease, anything. But he, poor thing, can’t even feed himself.*” (FM1); as had healthcare professionals: “*They often have unrealistic expectations, namely that a person falls ill and gets better.*” (N1).

A number of participants have **withdrawn from the future deterioration** of their health condition, not wanting to think about it or plan for it. Patients described it as follows: *“I don’t even want to admit these things.”* (P1)*; “I think I don’t even want to see it.”* (P10); “*I can’t imagine it at all. Maybe I don’t want to.”* (P9).

They prioritized the present: *“I’m dealing with today. I’m really afraid to deal with tomorrow or the day after tomorrow.”* (P9). They used this emphasis on the present as a defense mechanism: *“I’ve started to deal only with today. I don’t think about what tomorrow will bring, because I could go mad from it. As his condition is getting worse … every month.”* (P1). The process of withdrawal is clear in this description of communication between partners: *“I didn’t talk about it with my husband. He told me not to worry about it, that it would turn out OK somehow.”* (FM3).

### Quality of life

Patients or their family members who have come to terms with the disease stated that patients can have good quality of life: *“I think he has a relatively good quality life, in the circumstances.”* (FM1); *“The quality of life is relatively good, in the circumstances.”* (P7). A caregiver for a patient with HD remembered pleasant moments during social activities: *“She always loved to go there ... She was happy, she was laughing so much. Life was good for her at the time.”* (FM4).

On the other hand, patients who had not come to terms with the disease described their quality of life negatively: *“No future, no quality of life.”* (P2).

## Discussion

This study provides insight into how the extent of the information on patients´ health conditions and the way patients with chronic progressive neurological disease learn this information from doctors affect their adaptation to their disease. It was determined that adequate information about patients’ health status, and the use of coping strategies are related to their adaptation to their disease, and consequently, to their quality of life. The participants often considered the extent of the information provided, and the way they were informed to be inadequate, especially information about receiving the diagnosis, the progression of the disease, and the end of life. Our findings also confirmed that the adaptation to disease is a process that is significantly related to how patients with PND experience quality of life.

The patients, family members, and healthcare professionals in our study agreed that the information given to patients by neurologists is mostly inadequate, and frequently almost nonexistent; similar results have been reported in previous studies [4, 5, 17]. This also applies to the moment when the patient is informed about their diagnosis [18, 19]. Solari et al. [4] found that patients with MS wanted to be informed about the disease clearly and unambiguously. Information about the disease at the time of diagnosis gives patients early control over their disease. Adequate information and healthcare professionals’ emotional support is crucial to how patients with MS later cope with their disease [3, 6].

Neurologist report that they find delivering bad news, particularly about progressive deterioration in condition, and death and dying, very stressful and upsetting. Other studies confirm that breaking bad news is an emotional issue for doctors [20, 21]. Informing patients about their diagnosis is considered to be especially stressful [4], as is providing information about the progression of a disease, the terminal stage of a disease, or about death and dying [22, 23]. Neurologist must carefully consider whether, when and how to share prognosis information with patients [24]. Patients and their family members can vary in their ability to speak and communicate about their condition and prognosis [25]. Doctor-patient communication about end-of-life care planning is an important topic in palliative care. In the past ten years, there has been discussion regarding the possibility of linking palliative and neurological care [2, 7, 26–28]. Early palliative care may be appropriate for patients with PND [29, 30]. Before advanced stages of the disease are reached, patients and neurologist can talk about the progression of the disease, and create an end of life care plan, in accordance with patients’ wishes. Buecken et al. [31] and Knies et al. [32] found that patients with MS were willing to talk about topics related to death and dying. The rationale behind early communication is mainly that a large proportion of patients with PND will develop cognitive disorders in advanced stages of the disease. In some types of MS, PD, or MND, the progression may be very rapid. There can be enormous prognostic uncertainty in neurological diagnoses, with few validated prognostic markers [29]. Oliver et al. [30], on the basis of an analysis of scientific evidence, suggest that communication with patients with PND and their families should be open, and should include the therapeutic aim and available options (Level of evidence C). Communication may be affected by changes in the patients’ cognitive deficits and ability to speak. All physicians, including neurologists, should be familiar with basic palliative care skills, including the communication of bad news, and advance care planning [5].

Our research has shown that doctors have inadequate communication skills regarding the delivery of bad news. Currently, there are various tools available that can be useful to doctors, e.g., SPIKES (**S**etting up the interview, assessing the patient’s **p**erception, obtaining the patient’s **i**nvitation, giving **k**nowledge, addressing **e**motions, **s**trategy and **s**ummary) [33]. It is also appropriate to use the SPICT™ (Supportive & Palliative Care Indicators Tool), which identifies patients suitable for palliative care and contains recommended questions for advance care planning [34, 35]. Strupp et al. [36] state that a palliative care doctor may support primary care providers in communication with the patient about disease progression.

We also tried to find out the relationship between the patients´ adaptation to their disease and the information they were given. Knowledge and awareness of the disease helped patients adapt and gradually cope with the situation. Eklund and MacDonald [37] discovered that almost 60% of patients with MS reported feeling that they had needed professional psychological help at some point following their MS diagnosis, and about 30% reported that they were not coping well and currently needed professional help. Due to the varying rapid progression of each type of PND, the life span of each patient is different, often lasting for many years. Bodily attentiveness becomes an integral aspect of the experience of illness. With permanent physical impairment, one must compensate daily for the body’s disabilities and explicitly allow for its limitations. Even when there are lengthy periods of remission, the chronically ill remain uneasily attuned to the way the body feels and moves – always “on guard” for signs of an impending recurrence [38].

The interviewed patients also mentioned problems with coming to terms with their disease. The use of various coping strategies and adequate information about their state of health has helped the patients in our study to accept their disease. Participants mentioned external and internal coping strategies. Chronster and Chan [39] place a sense of self-mastery, control, self-efficacy, and interpersonal skills among internal coping resources, and social support and material goods among external ones. Holland et al. [40] found the most commonly coping strategies in patients with MS Acceptance, Active Coping, Planning and Positive Reinterpretation and Growth. Coping strategies have been identified as an important predictor of quality of life in patients with MS [41, 42].

Furthermore, some patients from our research clearly deny the reality of their disease. Patient denial prevented some from contact with other patients with the same disease, and with patient organizations, both of which can be a useful source of help. The denial of the disease has also prevented some patients from preparing for the future and planning adequate care. Joy and Johnston [38] consider the denial of the disease as one of the form of coping strategies which can focus either on problems or emotions. Emotion-focused strategies generally involve denial, and escape or avoidance, or reconfiguring the problem to make it more positive [38]. Studies on other diseases suggest that patients who use problem-focused techniques make better adjustments and have better outcomes than those who use avoidance and denial [36]. Emotion-focused methods predominate when patients sense things are beyond their control [43]. It may be beneficial to conduct research into this area for patients with PND.

Participants´ statements in our research also exhibited a sense of hopelessness and of not having come to terms with the disease. Hopelessness is one of the symptoms of depression. Cwastiak et al. [44], on the basis of an epidemiologic study of a large community sample, state that clinically significant depressive symptoms were found in 42 patients with MS. Patients with advanced disease and shorter duration of disease were much more likely to experience clinically significant depressive symptoms. In addition, Rickards [45] states that depressive syndromes in chronic neurological illness are common and disabling.

The results of our study also indicated that adaptation to the disease (coming/not coming to terms with the disease) was related to assessment of quality of life. Participants who had not yet come to terms with the disease described their quality of life as unsatisfactory. Bishop [46] points out that if chronic disease adversely affects patients’ quality of life, they can cope with their situation either by changing the importance of the domains of quality of life (values), by changing their perception of control over the domain of ​​quality of life, or by coming to terms with life with reduced quality. Kern and Brown [47] found that disease adaptation may have decreased quality-of-life responsiveness in patients with chronic PND. Also coping strategies used by patients with MS play a key role in adjusting to the disease and affect patient’s overall quality of life. Farran et al. [48] found that patients with MS using positive coping strategies had significantly higher scores of quality of life and lower depression. Not coming to terms with the disease, hopelessness, and depression negatively affect the perceptions of quality of life. This has been confirmed in research by Janardhan and Bakshi [49]. In the previous studies, depression was confirmed as a significant predictor for balance impairments in individual with MS [50] and significant predictor of low quality of life in all neurological diseases [47, 49, 51]. The participants in our study described depression when all their coping rexources had been exhausted. Many psychosocial factors including coping, mood, self-efficacy, and perceived support, influence the quality of life of patients with MS more than biological variables such as weakness [52]. For this reason, care of patients with PND requires a multidisciplinary team. Oliver et al. [30] recommend that care be provided by a multidisciplinary team consisting of at least three different professions: physicians, nurses, and social workers or psychologists/counsellors.

### Strengths and limitations

The use of multiple qualitative methods (individual interviews and focus groups) and data coding by more than one person ensured the validity of our approach. A strength of this study is that it described the experiences of a wide range of health care professionals from several settings, patients and their family member. Due to the complexity of the issue, the various specialist physicians (neurologists, physiatrists, and palliative care doctors), family members and health workers were included in the sample, leading to theoretical saturation of the sample. The low number of physicians (*n* = 6) represented in the sample may be a limitation of this study. Despite this limitation, the findings of this study do point to ways of improving the quality of care (better informing patients with PND about their disease by physicians).

### Implications

Our study highlights that all physicians, including neurologists, should be familiar with basic palliative care skills, including the communication of bad news, and advance care planning. It is beneficial to use open questions, to assess patients’ views on the amount of information they require, and to support patients in expressing their emotions when they receive bad news, as well as to support them in finding effective coping strategies. Informal meetings of patients with doctors and other health professionals, organized by patient organizations, can be an appropriate setting for learning more information about the disease, and receiving support. Informing patients with PND of their health status and help them with the adaptation to the disease needs effective collaboration between various professionals (neurologist, psychologists, nurses, and social workers), patients and their family members. Our findings may be useful for policy makers to understand the patients with PND and their family members in order to design adequate health systems.

For further research, we recommend verifying the impact of good communication skills of neurologist to better adaptation to the disease of patients with PND and increasing their quality of life in an intervention study.

## Conclusion

Good communication skills, which are integral to the development of a meaningful and trustworthy doctor-patient relationship, are indispensable for neurologists. Adequate information, support from healthcare professionals, and the use of effective coping strategies help the patient to adapt more effectively to the disease, and thus improve their quality of life.

## Data Availability

The datasets generated during and analyzed during the current study are not publicly available, due to the fact that the participants only provided informed consent to use of the data for the current study.

## Abbreviations

ALS: amyotrophic lateral sclerosis
Ergo: ergotherapist
FM: family members
HCh: hospical chaplain
HD: Huntington’s disease
MD: medical doctor
MMSE: Mini Mental State Examination
MND: motor neuron disease
MS: multiple sclerosis
N: nurse
P: patient
PD: Parkinson’s disease
Phys: physiotherapist
PND: progressive neurological diseases
Psy: psychologist
SPICT™: supportive & palliative care indicators tool
SPIKES: Setting up the interview, assessing the patient’s perception, obtaining the patient’s invitation, giving knowledge, addressing emotions, strategy and summary
SW: social worker
